# An artificial intelligence-based navigation system for enhancing anatomical recognition and safety during laparoscopic cholecystectomy: a pilot study

**DOI:** 10.1097/JS9.0000000000005086

**Published:** 2026-04-09

**Authors:** Keita Sonoda, Yuta Abe, Yutaka Nakano, Masashi Takeuchi, Yosuke Uematsu, Tasuku Furube, Minoru Kitago, Yasushi Hasegawa, Shutaro Hori, Masayuki Tanaka, Hirofumi Kawakubo, Yuko Kitagawa

**Affiliations:** Department of Surgery, Keio University School of Medicine, Tokyo, Japan

**Keywords:** acute cholecystitis, artificial intelligence, bile duct injury, laparoscopic cholecystectomy, surgical navigation

## Abstract

**Background:**

Failure to incise ventral to the Rouvière’s sulcus and maintain the dissection plane on the gallbladder (GB) surface predisposes patients to bile duct injuries (BDIs), particularly during trainee-performed laparoscopic cholecystectomy (LC). No existing artificial intelligence (AI) tools offer Tokyo Guidelines 2018 (TG 2018)-anchored real-time guidance. We developed and externally validated an AI navigation system that highlights the alert zone (AZ) – the hepatoduodenal-ligament tissues lying below an imaginary line from the roof of the Rouvière’s sulcus to the base of segment 4 and the infundibulum–cystic duct junction – and GB surface specified in the TG 2018.

**Materials and Methods:**

Seventy-three LC videos (January 2022–March 2024) were used to train the DeepLab v3 + segmentation model. The AZ and GB surface were manually annotated. The performance was tested on 10 independent videos (100 frames) with an intersection-over-union (IoU) metric. A two-arm pilot usability study randomized 10 fifth- or sixth-year postgraduate surgeons to answer video-based safety questions with or without AI assistance (20 tasks each).

**Results:**

The AI achieved a mean IoU of 0.703 (AZ) and 0.735 (GB) compared to the developer ground truth and 0.706 (AZ) and 0.730 (GB) compared to the external ground truth. With AI navigation, the correct selection of a safe incision point increased from 58% to 90%, and contour recognition of the GB surface increased from 70% to 92% (both *P* < 0.05).

**Conclusion:**

The AI navigation system based on the TG 2018 reliably delineated critical landmarks and markedly improved intraoperative trainee decision-making. Prospective real-time trials should determine whether this technology reduces BDIs.

## Introduction

Laparoscopic cholecystectomy (LC), the treatment of choice for gallbladder (GB) diseases, including acute cholecystitis (AC), was performed in 118 825 patients in Japan in 2023[1]. Although the National Institutes of Health consensus concluded that LC is a safe and effective treatment procedure[2], bile duct injury (BDI), one of the most critical complications of LC, has been reported to occur in 1.5% of cases[3]. BDI significantly affects healthcare outcomes, often requiring extended hospital stays and involving complex surgical or endoscopic interventions for repair in some cases[4]. Mortality rates associated with BDIs, although variable, are considerably elevated in severe cases, particularly when accompanied by postoperative sepsis or liver failure[5]. Furthermore, impaired activities of daily living following BDI can necessitate long-term rehabilitation or caregiving support, imposing additional burdens on both patients and healthcare systems. From an economic perspective, BDI management is associated with substantial costs, including those related to extended hospitalization, repeated interventions, and long-term monitoring^[^6,7^]^.

**HIGHLIGHTS**

- The developed AI navigation system enhances surgical performance during laparoscopic cholecystectomy (LC).
- The AI navigation system is expected to reduce the incidence of bile duct injury during LC.
- Our tool will be especially useful to trainees for improving the identification of safe incision points while consistently adhering to guideline-recommended dissection planes.

To prevent BDI during LC, the Tokyo Guidelines 2018 (TG 2018), a clinical guideline that has been widely adopted and evaluated worldwide^[^8–11^]^, advocated safe steps for LC to treat AC. These safe steps include “starting dissection from the posterior leaf of the peritoneum covering the neck of the GB and exposing the GB surface ventral to Rouvière’s sulcus (RS)” and “maintaining the plane of dissection on the GB surface throughout LC”[12]. In addition, TG 2018 proposed that any surgical procedure during LC should be performed ventral to the imaginary line between the roof of the RS and the base of segment 4 of the liver (S4)[12]. However, these steps are occasionally challenging, particularly in the presence of inflammation or fibrosis.

Artificial intelligence (AI) has dramatically changed medical imaging and video analysis in surgery and endoscopy, including intraoperative phase recognition and cancer detection^[^13–23^]^. The development of automated recognition systems for anatomical structures has also been reported for various surgeries. We also established recognition systems for upper gastrointestinal surgeries such as esophagectomy or gastrectomy and validated the clinical efficacy of these AI systems in our previous study. Our AI systems may be easily applicable to LC because important landmarks that have already been proposed by well-validated guidelines were annotated based on our previous experiences. Furthermore, AI navigation for LC plays a more significant role than for other surgeries because LC is mainly performed by trainee surgeons rather than experts in emergency settings.

For instance, several AI systems have been developed to recognize anatomical structures around Calot’s triangle^[^24–33^]^, while others have focused on identifying safe (“go-zones”) and dangerous (“no-go zones”) areas of dissection based on subjective annotations rather than defined anatomical landmarks^[^34–36^]^. However, no study has investigated the clinical efficacy of AI navigation for LC.

This study aimed to develop AI navigation for real-time anatomical recognition of the GB surface and alert zone (AZ), areas that require attention to dissect, potentially enhancing surgical safety and reducing the risk of BDIs. We also evaluated the clinical usefulness of this AI system by recruiting surgical trainees. This study is compliant with the TITAN Guidelines 2025[37].

## Materials and methods

### Datasets

We retrospectively selected surgical videos of a consecutive cohort of patients who underwent LC between January 2022 and March 2024. Patients who underwent cholecystectomy with liver bed resection, subtotal cholecystectomy, or had moderate or severe cholecystitis based on the TG 2018 criteria were excluded. As a result, a total of 83 patients were included. All procedures were performed by six board-certified surgeons, each recognized as an “Expert Surgeon” by the Japanese Society of Hepato-Biliary-Pancreatic Surgery and/or as a “Certified Endoscopic Surgeon” by the Japanese Society of Endoscopic Surgery. Patient characteristics, including age, sex, and clinical findings, were retrospectively evaluated. The surgical videos and corresponding annotations used in this study were collected from our institution and are not publicly available due to patient privacy regulations. The study protocol was approved by the Ethics Committee. The study was conducted in accordance with the ethical standards of the Declaration of Helsinki 1975. This study utilized a surgical video database compiled under an institutional review board-approved protocol. As part of standard hospital procedure, all patients were informed that their anonymized clinical data, including surgical videos, may be used for research purposes. Patients were provided with clear information and had the right to refuse (opt-out) participation at any time without affecting their care. This study included data from patients who did not opt-out. Ten trainee surgeons participated in the usability study, in which they performed anatomical identification tasks based on surgical videos with and without AI assistance. Verbal informed consent was obtained from all participating trainees prior to their involvement; this less stringent approach was implemented in line with the study being non-invasive, thereby not impacting clinical decision-making or patient care.

### Development of an AI algorithm

Seventy-three surgical videos were used to develop the AI model. From each video, an expert surgeon extracted 5–25 images in which the target anatomical structures were clearly identifiable at intervals of at least 10 s, excluding those that were blurred, obscured by mist, or distorted by water droplets.

**Figure 1.**
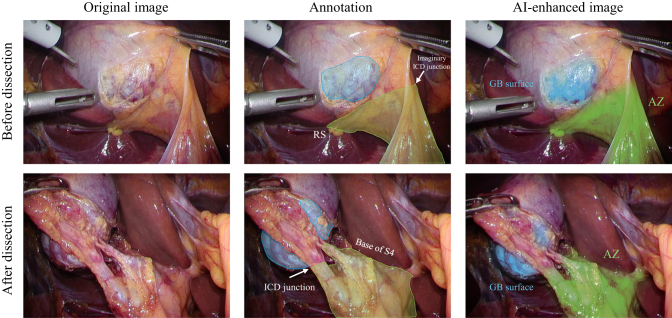
Examples of original, annotated, and AI-enhanced images before and after dissection. The superior border of the AZ is defined by a line connecting the RS, base of S4, and ICD junction. AI, artificial intelligence; RS, Rouvière’s sulcus; AZ, alert zone; ICD, infundibulum-cystic duct.

These images were annotated to identify the contours of the GB surface and AZ, defined as the hepatoduodenal ligament below a line connecting the roof of the RS, base of S4, and the infundibulum-cystic duct (ICD) junction (Fig. 1). On the image before dissection, the superior border of the AZ was drawn according to the imaginary ICD junction, which was based on information, including the image of the GB after completing the dissection was completed. The GB surface was defined as the wall structure within the fatty tissue that appeared when the GB serosa was dissected. Annotations were independently performed by two board-certified surgeons, and an expert surgeon verified the final annotations to ensure accuracy. All modeling procedures were performed using a script written in Python 3.7. The AI model training was conducted on a computer equipped with an NVIDIA GeForce RTX 3090 graphics processing unit (NVIDIA; Santa Clara, CA, USA) and an Intel Core i9-10900X central processing unit running at 3.70 GHz with 128 GB of RAM. The DeepLab v3 Plus framework was employed for semantic segmentation, which is a computer vision process that labels each pixel of an image to identify and outline different objects. An example of AI-enhanced videos of LC is shown in the Supplemental Digital Content (Video 1), available at: http://links.lww.com/JS9/H209.

### Evaluation of AI performance

The remaining 10 surgical videos were used to evaluate the AI model. Ten frames were randomly selected from each video for analysis. The performance of the segmentation algorithm was assessed using the intersection over union (IoU) metric, which measures the overlap between the AI-generated segmentation mask and ground truth data annotated by an expert surgeon who developed this navigation system. Additionally, for external validation, we assessed the IoU between the AI-generated segmentation mask and the data annotated by another expert surgeon who was not involved in the development of this navigation system. An IoU score of 1 indicates perfect alignment, whereas a score of 0 indicates no overlap.

### Usability of AI for surgeons

**Figure 2.**
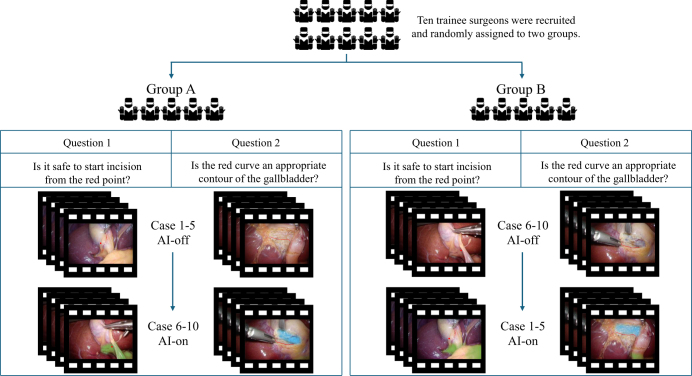
Overview of the experiments on the usability of AI Ten-second videos are created by an expert surgeon. Questions based on the last image of the movie in each experiment are asked to the trainee surgeons after they watched the movie. The recruited trainee surgeons are divided into two groups. AI, artificial intelligence.

To evaluate the usability of AI navigation in clinical practice, we conducted two experiments to compare the performance of surgeons with and without the assistance of AI navigation (Fig. 2). The same 10 surgical videos used to evaluate the AI model were also employed for this assessment, with 10 trainee surgeons in their fifth or sixth year of postgraduate training participating in the study. The surgeons were randomly assigned to two groups: Groups A and B. The experience of the participating trainees was comparable, with a median of 64 (range 36–110) LCs performed in Group A and 81 (range 34–105) in Group B. Group A watched the original surgical videos for cases 1–5, followed by the AI-enhanced videos for cases 6–10. Group B watched the original videos for cases 6–10 followed by the AI-enhanced videos for cases 1–5. The AI navigation system is designed as an assistive tool to augment, not replace, the surgeon’s clinical judgment. The surgeon retains ultimate responsibility for all intraoperative decisions.

## Experiment 1: Verification of the AZ as a landmark for starting an incision ventral to the RS

In this experiment, we evaluated the assistance of the AI in guiding the initial incision ventral to the RS. The expert surgeon extracted 10-s clips from the surgical videos immediately before the first GB incision. Ten original videos were prepared along with corresponding questions regarding the safety of the incision point in the final frame of each video. Additionally, we created 10 AI-enhanced videos to match the original videos. The videos and questions used in the experiment are provided in the Supplemental Digital Content (Video 2), available at: http://links.lww.com/JS9/H210.

## Experiment 2: Verification of the contour of the GB surface

This study focused on the ability of AI to help surgeons identify the contours of the GB surface. The expert surgeon selected 10-s clips from the dissection phase of each surgical video. Ten original videos were prepared with corresponding questions regarding the visibility and clarity of the GB surface contours in the final frame of each video. Ten AI-enhanced videos were also created to match the original set. The videos and questions used in this experiment are available in the Supplemental Digital Content (Video 3), available at: http://links.lww.com/JS9/H211.

### Statistical analysis

The accuracy rates were compared between groups assisted with and without AI navigation. A *t*-test was performed using JMP software (ver17.2.0), and a p-value of < 0.05 was considered statistically significant.

## Results

### Patient characteristics

The characteristics of the 10 patients in the test dataset are presented in Table 1.

**Table 1:** Patient characteristics.

	Test set (*n* = 10)
Age (year), mean ± SD	63 ± 15
Male	4 (40%)
Body mass index (kg/m^2^), mean ± SD	23.9 ± 3.2
Diagnosis	
Cholecystolithiasis	8 (80%)
Acute cholecystitis	2 (20%)
Operative time (min), mean ± SD	58 ± 24
Blood loss (mL), mean ± SD	9 ± 6

SD, standard deviation

### Evaluation of AI performance

The AI clearly shows the AZ in green and the GB surfaces in blue (Fig. 1). The mean IoU for the expert who developed this navigation system was 0.703 for the AZ and 0.735 for the GB surface. For the external validation, the mean IoU for the expert who was not involved in the development of this system was 0.706 for the AZ and 0.730 for the GB surface.

### Usability of AI for surgeons

Experiment 1: Verification of the AZ as a guide for starting the incision ventral to the RS

**Figure 3.**
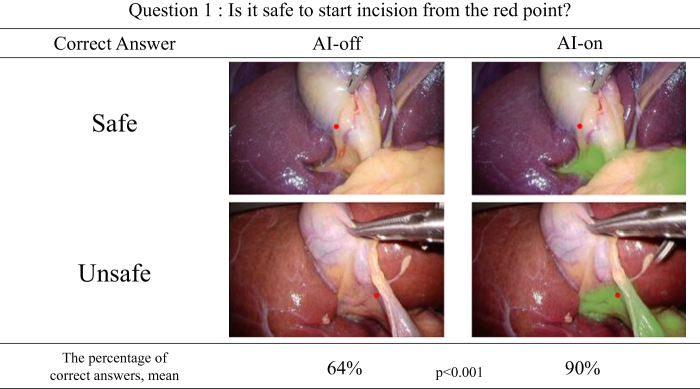
Result of experiment 1. Examples of questions on the AZ and percentage of correct answers AZ, alert zone.

Five trainee surgeons answered 10 questions based on the original videos of 10 cases. The percentage of correct answers was 58% for the original videos and 90% for the AI-enhanced videos (Fig. 3).

Experiment 2: Verification of the contour of the GB surface

**Figure 4.**
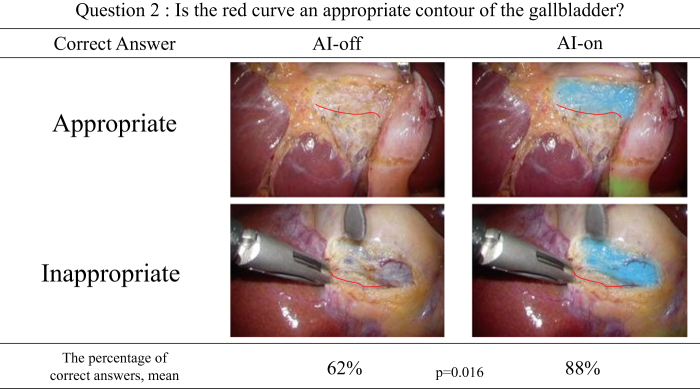
Result of experiment 2. Examples of questions on the GB surface and percentage of correct answers GB, gallbladder.

Five trainee surgeons answered 10 questions based on the AI-enhanced videos of 10 cases. The percentage of correct answers was 70% for the original videos and 92% for the AI-enhanced videos (Fig. 4).

## Discussion

This preliminary study demonstrated that trainee surgeons’ ability to identify key anatomical landmarks, as defined by expert consensus, was significantly improved with the use of our AI-based navigation system. This suggests that the system can enhance adherence to expert-defined safe dissection planes. To the best of our knowledge, this is the first report on an automated anatomical recognition system for LC based on TG 2018, a highly validated and widely adopted guideline for AC[8]. By focusing on the AZ and contour of the GB surface, our system supports two key TG 2018 recommendations: (1) initiating dissection ventral to the RS and (2) maintaining a plane strictly on the GB surface.

Several AI navigation systems for LC have been introduced; however, they have yet to focus on the safe steps of TG 2018. Mascagni *et al* and Tokuyasu *et al* proposed systems that navigate multiple anatomical structures related to LC^[^24,26,29,33^]^. However, these systems do not distinguish between the GB surfaces with and without serosa, which is a critical distinction for safely exposing the GB surface, as emphasized by TG 2018. Similarly, Madani *et al* developed an AI system based on subjective annotations of “go-zone” and “no-go zone” areas rather than relying on objective anatomical landmarks^[^34,36^].^ Tashiro *et al* reported an AI system capable of identifying loose connective tissues between the GB and surrounding tissues, a key area that becomes thickened, fibrotic, and mostly unidentifiable in cholecystitis[38]. The BDI risk was significantly higher in these cases[39]. By contrast, our AI system provides an anatomically grounded approach that was developed to help surgeons follow the safe steps of TG 2018. This navigation system emphasizes the AZ and GB surface, which supports surgeons in exposing the GB surface ventral to the RS and maintaining the dissection plain on the GB surface.

Recent studies have assessed the achievement of the Critical View of Safety (CVS) using AI^[^24,29,30^]^. However, these systems focus primarily on evaluating the CVS rather than providing real-time navigation to support surgeons during dissection before achieving the CVS. Our AI system addresses this gap by guiding surgeons before reaching the CVS, which is essential for preventing BDIs. Moreover, real-world data suggest that although the importance of achieving the CVS has been established, it is not always enforced in every case of LC. Nijssen *et al* reported that although CVS achievement was documented in 72% of cases based on operative records, a detailed video review revealed that true CVS was achieved in only 35.2% of cases[40]. This discrepancy highlights the need for real-time navigation systems to ensure safe dissection practices from the outset.

Indocyanine green (ICG) fluorescence provides real-time visualization of the liver and bile ducts[41]. While its primary application in LC involves visualization of the common bile duct (CBD), several reports have described the use of ICG to identify the GB surface^[^38,42^]^. However, there are notable limitations of ICG fluorescence in LC. First, visualizing the CBD does not necessarily aid surgeons, as they must avoid dissecting near this structure; hence, it was omitted from the safe steps in TG 2018. Second, imaging can be significantly impaired in patients with gallstones obstructing the cystic duct or in those with thickened GB walls due to inflammation. Third, ICG is contraindicated in individuals with iodine allergies and requires specialized equipment (ICG-specific cameras), thereby increasing overall costs. Finally, ICG fluorescence cannot distinguish between GB surfaces with and without the serosa, which is a critical consideration for maintaining safe dissection planes.

In the evaluation of the AI performance, the IoU compared to the ground truth annotated by one of the developers was 0.703 for the AZ and 0.735 for the GB surface. When compared with annotations by independent experts not involved in the development, which served as external validation, the IoU was 0.706 for the AZ and 0.730 for the GB surface, demonstrating comparable performance. While the achieved mean IoU demonstrates good concordance with expert annotation, the threshold for clinical reliability in high-stakes surgical navigation has not been established. These values indicate a strong assistive capability, but further model refinement and prospective studies are necessary to determine whether this accuracy level is sufficient to consistently improve safety outcomes, especially in complex cases. The system should be regarded as a guidance tool, with the surgeon’s judgment remaining paramount.

In the experiments on the usability of AI for surgeons, we demonstrated that trainee surgeons using our AI system were able to identify the GB surface more precisely and determine safer initial dissection points than those without AI assistance. The accuracy rates for identifying the AZ and GB surface among surgeons without AI navigation assistance were surprisingly low at 64% and 62%, respectively, although the participating trainees had 5–6 years of postgraduate experience, and the median number of LC-experienced cases was 64 in Group A and 81 in Group B. These findings highlight a gap in the real-world understanding and implementation of the safe dissection steps in TG 2018. Furthermore, LC is often performed by less experienced surgeons in emergency settings, particularly in cases of acute cholecystitis, which further increases the risk of nonadherence to safe steps. Our decision to focus on trainee surgeons stems from this reality; although hepato-pancreato-biliary surgery specialists routinely manage complex hepato-biliary anatomy, the risk of BDI is often associated with a lower surgical volume, a characteristic more common in general surgical practice^[^43,44^]^. Therefore, supporting this broader group of surgeons is the primary goal of our system.

It is important to note that AI is not perfect and serves only as an aid; final decisions regarding the line of dissection and other critical steps are ultimately made by the surgeon. In this study, the accuracy rates for identifying the AZ and GB surface among surgeons using AI navigation were 90% and 88%, respectively, which, while significantly improved, did not reach 100%. These results confirm that the validation method used in this study aligns with the reality of clinical practice.

Our AI navigation system has the potential to reduce BDI rates and associated morbidities by improving the surgeons’ ability to recognize critical anatomical landmarks and adhere to safe dissection planes. Compared with AI systems developed for other surgical areas, such as the esophagus, stomach, and prostate gland, the need for AI navigation in LC is arguably more pressing because of the frequent involvement of less experienced surgeons. Additionally, the use of our AI system may increase the number of early LC procedures performed for acute cholecystitis, as recommended by TG 2018, which is often underutilized in real-world clinical practice, where conservative treatments such as antibiotics or percutaneous transhepatic GB drainage are more commonly employed.

A key priority for future research is to translate this system into a real-time intraoperative guidance tool and validate its clinical impact. This will begin with the technical integration of the AI model into the operating room’s laparoscopic video feed to provide a seamless, low-latency overlay. Subsequently, a prospective trial is warranted to assess the system’s real-world influence on surgical workflow, surgeon decision-making, and safety outcomes. A parallel focus will be on enhancing the model’s robustness for severe cholecystitis by significantly expanding the training dataset with challenging cases and employing tailored data augmentation to better simulate difficult surgical fields.

## Limitations

This study has several limitations. First, we intentionally excluded cases of moderate and severe cholecystitis from this initial study to first establish a baseline model performance. Future work will focus on improving the model for more difficult scenarios such as severe cholecystitis. The system’s utility in these cases, where it is most needed, remains to be proven. Second, this was a preliminary study with a small cohort of 10 trainee surgeons in the usability test, which limits the generalizability of the findings. Third, the system’s performance on rare anatomical variations remains untested and is an important area for future investigation. However, it should be noted that the TG 2018 principles on which our AI navigation is based are intended to provide a standardized safe dissection plane that is reliable regardless of such underlying anatomical variations. Fourth, the ground truth for both internal and external validation was established by a single expert; future studies should incorporate annotations from multiple experts to establish a more robust consensus. Fifth, the usability study was conducted in a controlled, offline setting using pre-recorded videos. This environment does not capture the dynamic pressures or cognitive load of live surgery, and the observed benefits may be an overestimation. Furthermore, while annotations were cross-verified by an expert, we did not formally calculate the inter-rater reliability (e.g., Cohen’s kappa) between the two initial annotators. A prospective trial is planned to measure changes in the surgical workflow and outcomes. Finally, it is important to note that the AZ is a concept we defined for this AI system based on established safety principles. While anchored in anatomical landmarks from TG 2018, the concept itself requires broader validation by the surgical community.

## Conclusion

In conclusion, we developed an AI navigation system that aligns closely with TG 2018 and meaningfully enhances surgical performance in LC. By highlighting the AZ and GB surface, our system may help surgeons, particularly trainees, adhere more reliably to guideline-recommended dissection planes, potentially decreasing the incidence of BDIs and improving patient outcomes. Further prospective investigations focusing on real-time applications and broader case mixes, including complex cholecystitis, are required to confirm these benefits and pave the way for widespread clinical adoption. Future usability studies should also incorporate additional metrics, such as the surgeon’s response time and decision-making confidence levels, to provide a more comprehensive evaluation.

## Data Availability

De-identified datasets generated and analysed during the present study are available from the corresponding author (e-mail: abey3666@gmail.com) on reasonable request and with permission of the Keio University School of Medicine Ethics Committee.

## Research registration unique identifying number (UIN)

This research was registered to Japan Registry of Clinical Trials (JRCT). The trial ID is jRCT1030250220. https://jrct.mhlw.go.jp/en-latest-detail/jRCT1030250220.

## Assistance with the study

We thank Editage (www.editage.com) for English language editing.
